# Multiscale model of regional population decline in little brown bats due to white‐nose syndrome

**DOI:** 10.1002/ece3.5405

**Published:** 2019-07-04

**Authors:** Andrew M. Kramer, Claire S. Teitelbaum, Ashton Griffin, John M. Drake

**Affiliations:** ^1^ Department of Integrative Biology University of South Florida Tampa Florida USA; ^2^ Odum School of Ecology University of Georgia Athens Georgia USA; ^3^ Odum School of Ecology and Center for Ecology of Infectious Diseases University of Georgia Athens Georgia USA

**Keywords:** disease model, little brown bat, metapopulation dynamics, *Myotis lucifugus*, plausible parameter sets, *Pseudogymnoascus destrucans*

## Abstract

1. The introduced fungal pathogen *Pseudogymnoascus destructans* is causing decline of several species of bats in North America, with some even at risk of extinction or extirpation. The severity of the epidemic of white‐nose syndrome caused by *P. destructans* has prompted investigation of the transmission and virulence of infection at multiple scales, but linking these scales is necessary to quantify the mechanisms of transmission and assess population‐scale declines.
2. We built a model connecting within‐hibernaculum disease dynamics of little brown bats to regional‐scale dispersal, reproduction, and disease spread, including multiple plausible mechanisms of transmission.
3. We parameterized the model using the approach of plausible parameter sets, by comparing stochastic simulation results to statistical probes from empirical data on within‐hibernaculum prevalence and survival, as well as among‐hibernacula spread across a region.
4. Our results are consistent with frequency‐dependent transmission between bats, support an important role of environmental transmission, and show very little effect of dispersal among colonies on metapopulation survival.
5. The results help identify the influential parameters and largest sources of uncertainty. The model also offers a generalizable method to assess hypotheses about hibernaculum‐to‐hibernaculum transmission and to identify gaps in knowledge about key processes, and could be expanded to include additional mechanisms or bat species.

## 1 INTRODUCTION

Since 2006, North American bat abundances have declined dramatically as a result of white‐nose syndrome (WNS) caused by the fungal pathogen *Pseudogymnoascus* (formerly *Geomyces*) *destructans (Pd)* (Blehert et al., 2009; Frick et al., 2015; Lorch et al., 2011; Minnis & Lindner, 2013; Warnecke et al., 2012). The disease, which has been documented in multiple temperate bat species (Langwig et al., 2012), negatively impacts bats via dehydration and excessive fat depletion by interrupting periods of torpor during hibernation (Bernard & McCracken, 2017; Meteyer, Barber, & Mandl, 2012; Reeder et al., 2012; Warnecke et al., 2012) and disrupting electrolyte balance (Cryan et al., 2013). Survival rates of infected bats vary by species and colony, and while there is evidence that resistance may be developing in some colonies (Langwig et al., 2017), the fungus is spreading across the continent and abundances of multiple species continue to decline (Frick et al., 2017; Pettit & O'Keefe, 2017). Spatial spread of *Pd* among hibernacula and overwinter bat mortality have primarily been studied independently, but susceptible bat population dynamics are likely to be highly dependent on the mechanisms linking these processes.

Although there has been substantial work investigating mechanisms of transmission within hibernacula (e.g., Hoyt et al., 2016; Langwig, Hoyt, et al., 2015; Langwig et al., 2017), and the spread of the fungus across space (Maher et al., 2012; O'Regan et al., 2015), no study to date has investigated both within‐hibernaculum and among‐hibernacula transmission dynamics simultaneously. Linking these scales is important, given that feedbacks between multiple scales can produce thresholds and complex dynamics that cannot be predicted at a single scale (Davis, Trapman, Leirs, Begon, & Heesterbeek, 2008; Peters et al., 2004). Thus, understanding both within‐hibernaculum transmission and among‐hibernacula spread, as well as the feedbacks between them, is important for accurately predicting the effects of WNS on bat populations.

Recent models of within‐hibernaculum dynamics have revealed multiple plausible mechanisms of WNS transmission. For instance, *Pd* transmission between bats can be either frequency‐dependent (Langwig et al., 2017; Reynolds, Ingersoll, & H. a. Barton., 2015) or density‐dependent (Meyer, Stevens, & Blackwood, 2016) depending on clustering behavior during hibernation (Langwig et al., 2012), with potentially dramatic implications for colony persistence (Frick et al., 2017; Langwig et al., 2012). In addition, *Pd* can persist for an extended period in the environment (Lorch et al., 2013; Reynolds et al., 2015), creating an environmental reservoir that propagates infection both within and between hibernation periods (Hoyt et al., 2015; Verant et al., 2018). Theoretical studies of the role of environmental persistence in driving WNS infection dynamics provide evidence that environmental persistence could increase WNS‐induced bat mortality (Meyer et al., 2016; Reynolds et al., 2015); however, because studies have not incorporated empirical data on bat populations, the importance of an environmental reservoir for bat population persistence is unclear.

Unlike within‐hibernaculum dynamics, models of between‐hibernacula spread of *Pd* have focused on describing general patterns of infection at broader (county‐level) spatial scales, rather than investigating specific mechanisms of spread (Maher et al., 2012; O'Regan et al., 2015). Accordingly, the mechanisms by which WNS spreads among hibernacula are not well understood. Bat‐to‐bat transmission is unlikely to occur during summer because bats clear signs of infection during nonhibernation periods (Langwig, Frick, et al., 2015). In addition, most bats consistently return to the same cave for hibernation (e.g., philopatry rates as high as 96% (Norquay, Martinex‐Nunez, Dubois, Monson, & Willis, 2013)), meaning that dispersal of *Pd* between caves is unlikely to be driven primarily by actively infected individuals entering uninfected hibernacula at the beginning of hibernation. Nevertheless, white‐nose syndrome has spread quickly through a network of patchily distributed hibernacula (Maher et al., 2012). Fall swarming is unlikely to result in transmission between bats (Langwig, Frick, et al., 2015), but the presence of *Pd* in the environment could be important during this phase, given that bats enter and leave multiple hibernacula during this period (Fenton, 2012; Norquay et al., 2013). Modeling this mechanism of among‐hibernacula spread alongside within‐hibernacula infection dynamics can help determine whether *Pd* could spread between caves during fall swarming.

The impacts of *Pd* can vary widely among hibernacula, where bat populations in some hibernacula go extinct, while others persist at reduced population sizes (Frick et al., 2017). A number of mechanisms could drive this variability, including the importance of density‐dependent transmission, tolerance, and resistance in different populations (Frick et al., 2017; Langwig et al., 2017). In addition to these mechanisms, metapopulation dynamics (e.g., rescue effects from uncontaminated hibernacula) could be important for sustaining populations infected by *Pd* (Grenfell & Harwood, 1997), but few studies have considered the role of interhibernacula dispersal in driving population persistence for the WNS system. Further, variability in environmental conditions among hibernacula could produce differences in WNS‐driven mortality, even without resistance or tolerance as an explanatory mechanism (Forsythe, Giglio, Asa, & Xu, 2018; Gargas, Trest, Christensen, Volk, & Blehert, 2009; Langwig et al., 2012). For instance, the growth rate of *Pd* on bats increases with temperature up 12–15°C (Verant, Boyles, Waldrep, Wibbelt, & Blehert, 2012)), leading to reduced survival in warmer portions of hibernacula (Langwig et al., 2012). Together, these results suggest that environmental variation among hibernacula (Hoyt et al., 2016; Langwig et al., 2012) could be a primary driver of differences in bat mortality rates (Hayman, Pulliam, Marshall, Cryan, & Webb, 2016).

Here, we report on a model we built that incorporates both explicit within‐hibernaculum transmission dynamics and among‐hibernacula dispersal for a single species, the little brown bat (*Myotis lucifugus*), which is spatially widespread and severely threatened by WNS (Frick et al., 2010; Maslo, Valent, Gumbs, & Frick, 2015; O'Regan et al., 2015). Using this model, we followed overwinter infection dynamics within multiple caves to estimate the number of bats that emerge each spring. We then linked these caves during summer, when bats congregate in maternity colonies and then disperse to caves again in the fall. During this fall swarming period, new hibernacula subpopulations could become infected by bats that visit infected caves and transmit *Pd* to a previously uninfected hibernaculum. We examined how this process affects infection and survival across multiple years. The model was parameterized using data on both winter survival and among‐hibernacula spread of WNS. Finally, we explored how among‐hibernacula heterogeneity in climate and the degree of philopatry on equilibrium might affect population sizes and population persistence.

## 2 METHODS

### 2.1 Within‐hibernaculum dynamics

We consider a model of within‐hibernaculum fungal growth and bat mortality, where susceptible little brown bats (*S*) can become infected with the fungus either from other bats or the environment; infected bats (*I*) decline in body condition into classes *F*_1_ and *F*_2_, and bats in class *F*_2_ die from disease (Table 1). Mortality from non‐WNS causes was implicitly modeled in the population growth phase (see below). Infected bats also shed the fungus, thus contributing to a hibernaculum's environmental reservoir. Previous models of WNS have incorporated multiple infection classes (Langwig et al., 2017; Meyer et al., 2016) and/or an environmental reservoir (Meyer et al., 2016), reflecting the importance of fungal load in determining mortality and the importance of environmental transmission. However, these previous models only included shedding or infection from bats in the most highly infected class (Meyer et al., 2016) or assumed no differences in bat‐to‐bat infection rates from bats with different fungal loads (Langwig et al., 2017), two ends of the spectrum of transmission mechanisms. In contrast, we included shedding and infection by all infected bats, with a parameter that reflected relatively lower shedding and infection rates by bats with lower fungal loads.

**Table 1:** Parameter ranges and sources

Parameter	Description	Scale	Units	Reported value	Tested range	Fit range	Source
ϕ	Environmental shedding	Within	CFUs Bats^−1^	50	12.5–200	16.8–186.5	Meyer et al. (2016)
ϵ	Relative shedding of heavily infected	Within	–	0.1	0–1	0.059–0.999	Meyer et al. (2016)
ψ	*Pd* growth in environment	Within	d^−1^	0.05–0.42	0–0.4	0.023–0.355	Reynolds et al. (2015)
κ	Loss of *Pd* from environment	Within	d^−1^	–	0.1	0.1	Meyer et al. (2016)
*β*_1_	Infection from the environment	Within	Bats^−1^ CFUs^−1 ^d^−1^	1 × 10^−12^	1e−13–1e−11	1.02e−13–9.71e−12	Meyer et al. (2016)
*β*_2_	Infection from infected bats	Within	Bats^−1 ^d^−1^	1.4e−4, 0.066^a^	1e−5–2.1e−1	1.20e−5–1.60e−1	Meyer et al. (2016); Langwig et al. (2017)
*γ*_1_	Transition to visibly infected	Within	d^−1^	1/80	1/100–1/50	0.0103–0.0200	Lorch et al. (2011)
*γ*_2_	Transition to heavily infected	Within	d^−1^	1/30	1/80–1/10	0.0130–0.0983	Lorch et al. (2011)
*μ*	Death from infection	Within	d^−1^	1/30	1/80–1/10	0.0132–0.0980	Lorch et al. (2011)
d	Gradient between frequency‐ and density‐dependent transmission	Within	–	–	0, 1	0, 1	–
*α*	Probability of avoiding infection from infected hibernacula	Among	–	–	0.9995–0.99999	0.9995–0.99996	–
*V*	Number of infected hibernacula visited during swarming	Among	–	–	1–14	1.697–13.20	–
λ	Population growth	Among	–	0.977–1.200	0.977–1.200	–	Frick et al. (2010)

Note

Fit range is based on the 33 parameter sets matching 6 goodness‐of‐fit statistics.

a Converted from 2 bats/month to 0.066 bats/day.

This system is represented by the following set of differential equations:

$$\frac{\mathrm{dE}}{\mathrm{dt}}=\phi \left(\epsilon I+F_{1}+F_{2}\right)-\kappa E+\psi E$$

$$\frac{\mathrm{dS}}{\mathrm{dt}}=-\beta_{1}SE-\frac{\beta_{2}S\left(\epsilon I+F_{1}+F_{2}\right)}{{\left(S+I+F_{1}+F_{2}\right)}^{d}}$$

$$\frac{\mathrm{dI}}{\mathrm{dt}}=\beta_{1}SE-\frac{\beta_{2}S\left(\epsilon I+F_{1}+F_{2}\right)}{{\left(S+I+F_{1}+F_{2}\right)}^{d}}-\gamma_{1}I$$

$$\frac{dF_{1}}{\mathrm{dt}}=\gamma_{1}I-\gamma_{2}F_{1}$$

$$\frac{dF_{2}}{\mathrm{dt}}=\gamma_{2}F_{1}-\mu F_{2}$$

where *E* is the environmental load of the fungus, and *S*, *I*, *F*_1_, and *F*_2_ are numbers of uninfected (susceptible), recently infected, moderately infected, and highly infected individuals, respectively (Langwig et al., 2017). The environmental contamination is produced by shedding of fungus from infected bats at a rate ϕ, where moderately and highly infected individuals (*F*) shed more than do recently infected individuals (*I*) (i.e.,ϵ < 1). The environmental *Pd* load changes as *Pd* dies at a rate κ and increases as *Pd* multiplies in the environment at the rate ψ. This environmental load (measured as colony‐forming units (CFUs) is quantified at the hibernacula scale, and insufficient information is available to consider localized differences in concentration on substrate. Susceptible individuals become infected at a given rate, *β*_1_, from environmental contact with the fungus, and at a different rate, *β*_2_, from contact with infected bats, where moderately and highly infected individuals are once again considered more infectious. Transmission between bats may be either density‐dependent or frequency‐dependent given the value of *d* (i.e., *d* = 1 reflects frequency dependence and *d* = 0 reflects density dependence). Once infection occurs, the fungus grows and the body condition of an individual declines, resulting in a transition to the more infected classes (*F*) at rates *γ*_1_ and *γ*_2_. Deaths of highly infected individuals occur at a rate *μ*.

Later, we varied these rates between caves to consider differences in transmission and mortality due to the environmental effects on pathogen dynamics (Table 1), extensions could use time‐varying versions of these parameters to account for development of resistance (Langwig et al., 2017).

We assumed initial infection in each hibernaculum proceeds from *Pd*‐infected bats (see below) (Verant et al., 2018), and then, in following years, bats could also be reinfected from the persistent environmental stage of the pathogen (Frick et al., 2017; Langwig, Hoyt, et al., 2015). We used constant parameter values in each hibernaculum over time, under the assumptions that the climates of individual hibernacula were consistent from year to year (Perry, 2013) and that transmission mechanisms remained constant over time. We must also make the simplifying assumption that *Pd* dynamics are driven solely by little brown bats, although hibernacula often include multiple species, this is necessary due to the fact that disease dynamics are best understood for this species and helped by the fact that they were the most abundant species and had the highest *Pd* load (Verant et al., 2018) in the hibernacula considered. Because the period of initial infection and eventual decline involves small numbers of individuals, we modeled the within‐hibernaculum dynamics as a Bernoulli process with stochastic rates determined by the model parameters (Table 1). This is in contrast to previous models with deterministic disease dynamics (Meyer et al., 2016; Thogmartin et al., 2013); we compared these results with a deterministic model of within‐hibernaculum dynamics using the same parameters to confirm the influence of stochasticity, given the additional computation required (see below).

### 2.2 Among‐hibernacula dynamics

At the end of winter, all living bats were assumed to emerge from hibernacula to depart for maternity colonies. At this point, they clear the fungus (Langwig, Frick, et al., 2015), and we made the assumption that all surviving individuals can be treated equally with respect to reproduction and dispersal, regardless of their infection class at the end of winter. These individuals reproduce and disperse to hibernacula with a fixed probability of returning to the cave occupied the previous winter. Baseline site fidelity was assumed constant at the best estimate of 96% (Norquay et al., 2013), and then, sensitivity to site fidelity was considered in an experiment with lower fidelity of 92% (Kunz, 1982) and perfect fidelity of 100%.

We model both reproduction and dispersal as stochastic events. Summer population growth rates λ were drawn randomly from a set of previously estimated possible values (Frick et al., 2010); these implicitly integrate sources of non‐white‐nose mortality and assume no carryover effects of possible WNS infection (but see Davy et al., 2017). We assumed that the metapopulation was at its carrying capacity prior to infection, so that if the selected growth rate would have resulted in a metapopulation size above the initial size, then another growth rate was selected (ceiling mechanism; Thogmartin et al., 2013). If no available growth rates could reduce the metapopulation size below carrying capacity, the size was set to carrying capacity. After reproduction, dispersal of non‐site‐faithful individuals occurred randomly to the other hibernacula in the system. We selected a uniform distribution for dispersal distances after comparing the fit of uniform, exponential, log‐normal, and power‐law distributions to the data from Norquay et al. (2013). Over longer scales, distance‐dependent dispersal is likely, given a significant effect of distance on the continental‐scale spread of WNS (Maher et al., 2012), but dispersal distances do not differ significantly from uniform over the scale of ~600 km (Norquay et al., 2013).

We modeled transmission of the fungus among hibernacula during the fall swarming period. Under this hypothesis, bats could enter multiple hibernacula during swarming before settling in a single hibernaculum for the duration of the winter. We represented the probability that a bat becomes infected during swarming as:

$$p_{\text{bat infected}}=1-{\left[\frac{U}{T}+\alpha \left(1-\frac{U}{T}\right)\right]}^{V}+$$

where *U* is the number of uninfected hibernacula, *T* is the total number of hibernacula, *α* is the probability a bat does not pick up infectious *Pd* when visiting an infected hibernaculum, and *V* is a parameter representing the number of different hibernacula entered during the swarming period. Recent detections of *Pd* on bats using caves for summer roosts (Ballmann, Torkelson, Bohuski, Russell, & Blehert, 2017; Carpenter, Willcox, Bernard, & Stiver, 2016) can be accommodated under this model; it would result in increased chance of entering an infected cave (*V*). Given limited published data on the tendency to visit different hibernacula, this expression makes two simplifying assumptions: equal probability of visiting any hibernaculum in the system and equal risk of picking up infectious *Pd* from any infected hibernaculum.

By dispersing the infected bats among hibernacula as described above, we produce a stochastic variable representing the number of infected bats returning to each hibernaculum at the beginning of winter. The probability of a previously uninfected hibernaculum *i* becoming infected is then:

$$p_{\text{hibernaculum i infected}}=1-{\left(1-p_{\text{bat infected}}\right)}^{N_{i}}$$

where *N*_i_ is the number of bats in the hibernaculum.

We also modeled fungal growth or persistence of the environmental reservoir of *Pd* during the summer (Verant et al., 2018), using the same rates as during the winter. For all hibernacula that were infected during the previous winter, their starting value in the next winter (*E*) is a function of the environmental *Pd* growth and decay rates, the size of the environmental reservoir at the end of the previous winter (*E*_0_), and the duration of the summer (*t*, which is equal in all simulations):

$$E=E_{0}e^{\left(\psi -\kappa \right)\left(t\right)}$$

### 2.3 Parameter estimation

Starting values for within‐hibernaculum parameters were collected from the literature (Table 1). Given that the structure of our model differs from the sources of these parameters and that coupling of the within‐hibernaculum and among‐hibernacula scales makes it difficult to use maximum likelihood to fit parameters, we used the method of plausible parameter sets (Drake et al., 2015) to identify parameter ranges that produce model outputs that replicate empirically documented patterns. Briefly, we produced 1,000 possible parameter combinations from the possible parameter values using Latin hypercube sampling (package "lhs" in R (Carnell, 2009)). Each of these sets was duplicated with a density‐dependent transmission version (*d* = 1) and a frequency‐dependent transmission version (*d* = 0), yielding 2,000 total combinations. We then ran 100 simulations of the model for each parameter set (i.e., for a total of 200,000 simulations). For parameter ranges that extended over several orders of magnitude (*β*_1_, *β*_2_, and *α*), we log‐transformed before the Latin hypercube sampling and then back‐transformed before running the simulations to achieve more representative sampling of variation.

Using these simulation results, we assessed the fit of results of each parameter set by comparing model results to eight statistical probes for which we had expected values from the literature. These metrics were the prevalence of infection at the end of year 1 (0.4–0.88, (Frick et al., 2017; Hoyt et al., 2018; Verant et al., 2018), survival at the end of year 1 (0.15, Langwig, Hoyt, et al., 2015), *Pd* abundance in infected hibernacula (10^6^–10^12^ CFU, Reynolds et al., 2015), the time to infection for all hibernacula in New York (O'Regan et al., 2015), and the number of newly infected hibernacula in New York in each year from 2 to 5 (O'Regan et al., 2015). The infection data from New York represent the best available information on infection times of individual hibernacula (as opposed to county level), despite being detection of disease rather than current methods of using DNA to detect *Pd* presence (Janicki et al., 2015). We also acknowledge that using the published lower limit of *Pd* prevalence of 0.4 in year 1 may not fully encompass this highly variable measure (K. Langwig, personal communication), but we use the published range as the best supported option and as one that is more likely to correspond to the type of hibernaculum‐level detection of disease in the New York dataset (compared to an alternative of allowing prevalence in year 1 of near zero). For each parameter set, we determined whether the expected value for each metric fell within the 95% quantile of values from simulations, then selected the parameter sets that performed best (i.e., those for which the most metrics jointly matched model predictions).

### 2.4 Simulation methods

We implemented both stochastic and deterministic versions of the within‐hibernaculum model to measure the effect of stochasticity on within‐hibernaculum and among‐hibernacula dynamics. By comparing stochastic and deterministic models, we can assess the influence that real‐world stochasticity may have on the overall dynamics of the system. In the deterministic version, we solved the within‐hibernaculum differential equations using the “lsoda” solver from package deSolve in R (Soetaert, Petzoldt, & Setzer, 2010). For stochastic simulations, we simulated within‐hibernaculum dynamics using an approximation of Gillespie's method (Gillespie, 1976) implemented using the “adaptivetau”package in R (Johnson, 2019). To increase computation speed, we modeled the environmental *Pd* abundance (*E*) deterministically, but bat infection classes stochastically. In both cases, we simulated 100 replicates for each of 2,000 parameter sets; these replicates were necessary for the deterministic implementation because we retained stochasticity in the among‐hibernacula model (i.e., in dispersal and reproduction).

Simulations were initialized to resemble the New York State population of little brown bats prior to the outbreak of WNS and run forward for 10 years. The New York State Department of Environmental Conservation reported year of disease detection for 54 little brown bat hibernacula (O'Regan et al., 2015) that were affected by WNS. For each of the 100 simulations, we created random initial colony sizes for these 54 hibernacula using a spline fit to the long‐tailed distribution of pre‐WNS hibernacula population sizes reported by Turner, Reeder, and Coleman (2011). These 100 initial population configurations were reused across the various parameter sets and experiments, and represent a wide range of initial population sizes.

### 2.5 Effects of philopatry and hibernaculum microclimate

We examined two biological and environmental characteristics with potential to influence the dynamics of the bat metapopulation. First, we examined how the tendency of bats to return to the same hibernaculum each year influences outcomes. For the case of bats dispersing more freely, we used a return rate of 0.92 from Kunz (1982), and for comparison, we assumed perfect philopatry with no dispersal. In both cases, we assumed no difference in swarming behavior (e.g., probability of visiting infected hibernacula during fall swarming). We also conducted an experiment examining the impact of potential variation in fungal growth rates between hibernacula. Cave microclimate is known to influence *Pd* growth (Forsythe et al., 2018; Martínková et al., 2018; Verant et al., 2012) and survival of infected bats (Langwig et al., 2012). Thus, we constructed a scenario in which the rate of disease progression to moderately infected (*γ*_1_) varies among hibernacula. We drew random *γ*_1_ values from a normal distribution with mean and variance equal to the mean and variance in *γ*_1_ from the most plausible parameter sets. This necessarily restricts the variation among hibernacula to the range of the parameter considered in the initial model simulations. We performed 100 simulations that differed in the random values of hibernaculum quality for each of the plausible parameter sets, using the same 100 initial population distributions as in the original simulations.

## 3 RESULTS

When the simulation results were compared to the observations from the literature, there were 42 sets of parameters (out of 2,000) with prediction intervals matching 6 of the 8 goodness‐of‐fit measures (Table S1 and Figure S1). Examining the range of model outcomes among all 2,000 parameter sets reveals several patterns. First, there is a positive relationship between the rate of transmission between bats (*β*_2_) and the prevalence of infection in the first year of infection in each hibernaculum (Figure S1). Second, the growth of fungus in the environment (ψ) strongly affects not only the quantity of *Pd* in the environment, but also prevalence and survival of bats in the second year of infection in a hibernaculum (Figure S1). Unsurprisingly, there are also clear effects of the among‐hibernacula transmission parameters (*α* and *V*) on the spread of WNS from hibernaculum to hibernaculum, where only parameter sets with relatively high risk of infection (i.e., low *α*) or relatively large numbers of hibernacula visited (i.e., high *V*) were able to match the observed rate of hibernacula infections in year 3 of the epidemic (Figure S1).

**Figure 1:**
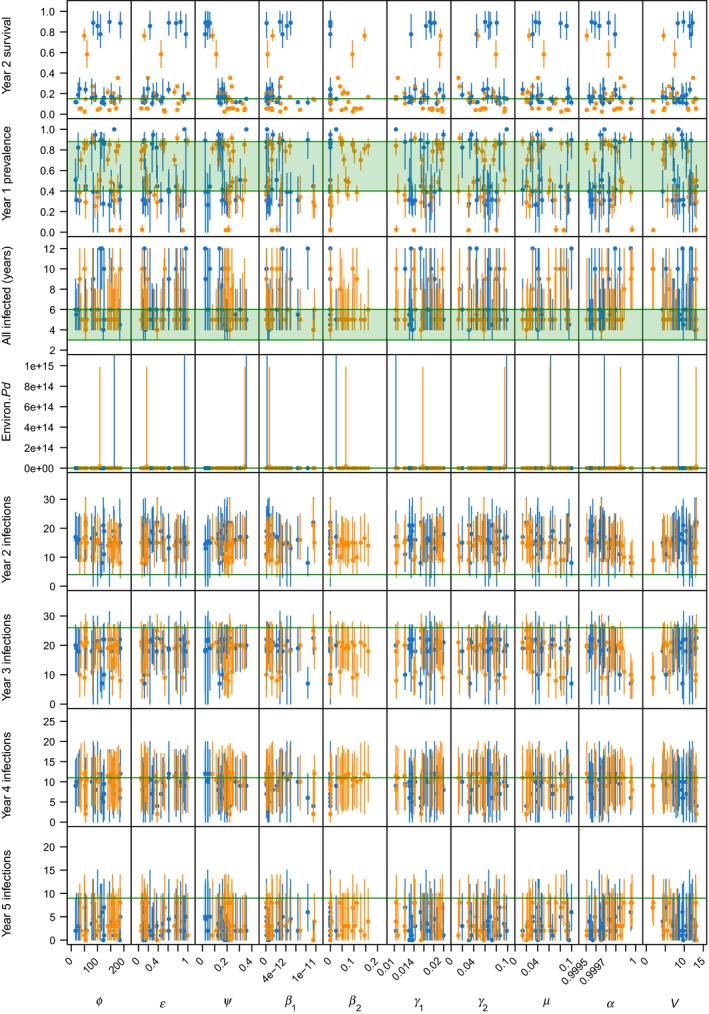
Match of model output with eight goodness‐of‐fit measures plotted against individual parameter values. Points are the median of 100 simulations of each parameter combination with the line representing the 95% prediction interval. Goodness‐of‐fit measures are in green. The 42 plausible parameters sets (matching 6 of 8 measures) were equally split between density‐dependent transmission in blue and frequency‐dependent transmission in orange

Testing all the parameter combinations under both density‐dependent and frequency‐dependent transmission revealed a clear difference between the plausible parameter values for the contrasting modes of transmission. While the number of sets matching 6 goodness‐of‐fit measures were equal at 21 for each transmission mode (Table S1), only very low rates of contact transmission (*β*_2_) were plausible under density‐dependent transmission, while a much wider range of values led to plausible outcomes when transmission was frequency‐dependent (Figure 1). If the pool of plausible sets is expanded to include those matching 5 goodness‐of‐fit measures, the pattern is similar, with density‐dependent transmission only plausible for low transmission rates (Figures S2 and S3). This distinction between transmission modes comes primarily from observed infection prevalence in bats; under density‐dependent transmission, prevalence of infection in the first year of infection rose rapidly with contact transmission (*β*_2_), and models had consistent problems matching the number of new hibernacula infected in year 2 of the epidemic (Figures S3). Frequency‐dependent transmission simulations included observed year 1 prevalence for a large range of transmission rates (Figures S3 and S4).

**Figure 2:**
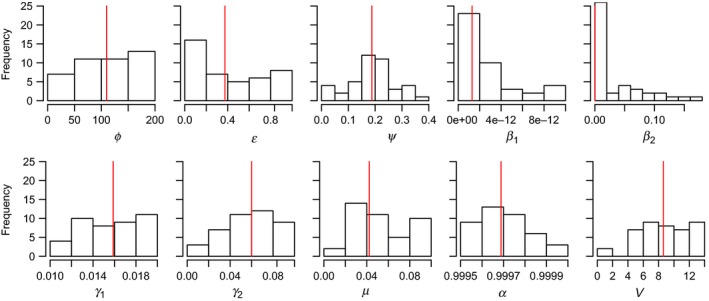
Histogram of the parameter values from the plausible parameter sets. Included are parameter values from all combinations that matched 6 of the 8 goodness‐of‐fit measures. Red lines indicate the median values

Closer examination of the 42 most plausible parameter combinations indicates that two measures were consistently the most difficult to capture: within‐hibernaculum survival in the year after infection and the number of new hibernacula infected in year 2 of the epidemic (Figure 1). The one validation metric where models with density‐dependent transmission were more likely to match real‐world observations was within hibernaculum survival, but this was offset but a higher rate of mismatches in year 3 and year 5 infections and in *Pd* count (Figure 1). Models tended toward a bimodal distribution of survival in year 2 (i.e., very few parameter sets predicted survival between 0.25 and 0.75); this was driven by the rate at which fungus proliferated in the environment (Figure 1 and Figure S3), where survival was lower when the fungus grew more quickly. Most distributions of the plausible parameter sets were relatively flat, except for contact transmission (*β*_2_), which tends toward lower values (Figure 2) because of the relationship with density‐dependent transmission discussed above and the bias in sampling caused by log‐transforming this parameter (see Methods). This log transformation also accounts for the apparent skew in transmission from the environment (*β*_1_). Notably, there is not a similar skew in *α*, the remaining log‐transformed parameter, suggesting that larger values are overrepresented in the plausible sets. Expanding consideration to the larger pool of plausible parameter sets (i.e., those that matched at least five validation metrics) confirms these patterns, as well as a tendency for intermediate values of *Pd* growth in the environment (ψ) (Figure S5).

The wide ranges we found for most parameters could be due to trade‐offs between pairs of parameters. However, after accounting for multiple testing, the only two strong correlations detected were a negative relationship between the among‐hibernacula parameters *α* and *V* (*r* = 0.44) and transmission mode and *β*_2_, where density dependence was associated with lower values of *β*_2_ (*r* = 0.65; Figure S6).

**Figure 3:**
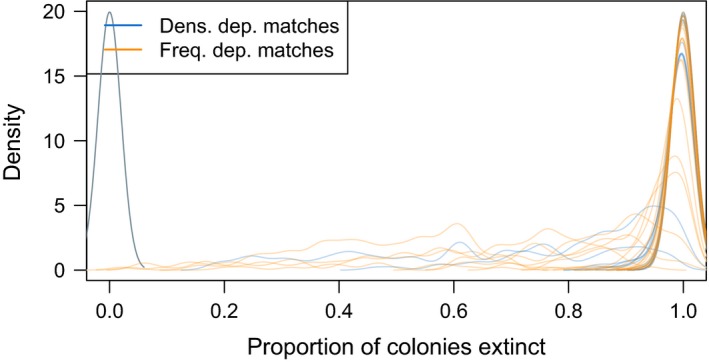
Distributions of the proportion of hibernacula without bats at the end of the simulation for the plausible parameter sets. The density‐dependent parameter sets are blue, and frequency‐dependent transmission is in green

The 42 parameter sets that matched 6 of 8 measured outcomes displayed a range of simulated outcomes (Figure 3, Figure S7 and S8). Most exhibited rapid decline in population sizes in the first three years (Figure S7) but diverged after year 3. This divergence resulted in high variation in the proportion of hibernacula harboring zero bats by the end of the 10‐year simulation period (Figure 3). However, even though some simulations predicted low hibernaculum‐level extinction, many of these persistent colonies consisted of only a handful of individuals. A subset of density‐dependent transmission were exceptions to this pattern, showing stable population and no extinctions (Figure S7 and Figure 3). These are simulations where the infection failed to persist when it started in colonies with small numbers of bats. The number of hibernacula newly infected in each year was variable even within individual parameter sets, but generally peaked shortly after initial infection (Figure S8).

### 3.1 Role of stochasticity in within‐hibernaculum dynamics

**Figure 4:**
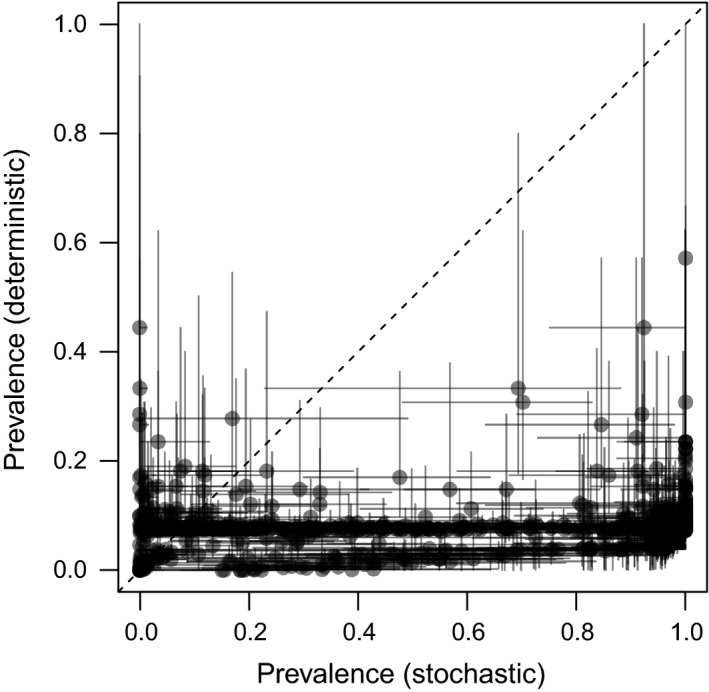
Correspondence in hibernaculum‐level prevalence in first year of infection between stochastic and deterministic simulations for each parameter combination. Dots are medians, and lines are the 95% prediction intervals. Deterministic simulations were deterministic for the within‐hibernaculum but not the among‐hibernacula model; variation in deterministic simulations comes from stochasticity in the among‐hibernacula model, including variation in the timing and order of cave infection

Including stochasticity in the processes of infection and disease progression greatly altered the range of possible outcomes. Compared to a version of the model with deterministic within‐hibernaculum dynamics, prevalence of infection was on average higher and more variable with stochasticity (Figure 4). There were large differences in survival between stochastic and deterministic simulations in many cases, but high variability in the direction of this effect (Figure S9). Hibernaculum‐to‐hibernaculum spread tended to be faster in the deterministic model, despite the fact that the spread between caves was still stochastic in nature, reinforcing that within‐hibernaculum dynamics are important not only to the number of bats remaining at the end of winter, but also for transmission among hibernacula (Figure S10).

### 3.2 Effect of changes in dispersal and hibernaculum quality

**Figure 5:**
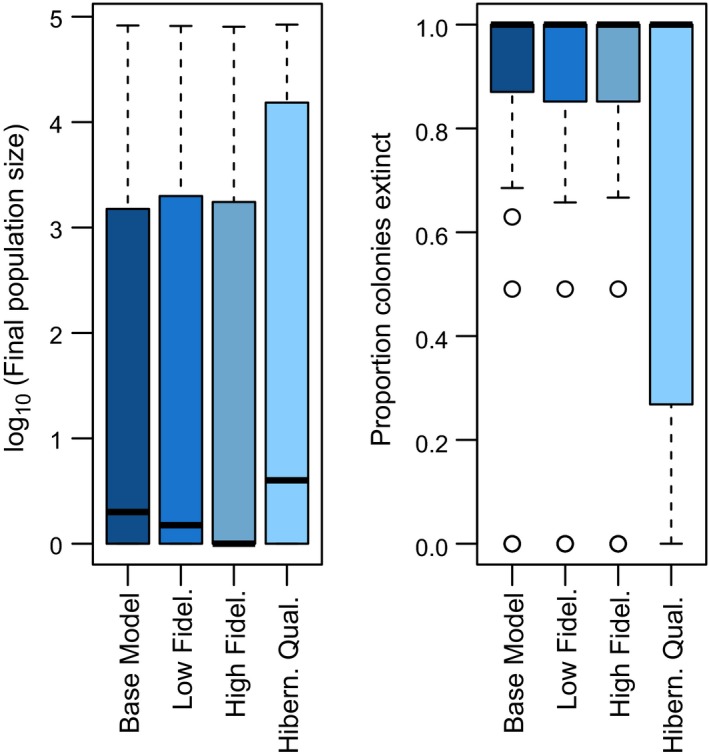
Boxplot showing effects of philopatry and hibernacula quality on median metapopulation size in year 10 and proportion of hibernacula lacking bats. The base model is compared to a low‐fidelity (philopatry = 0.92) and high‐fidelity (philopatry = 1) scenario. Hibernacula quality represents the introduction of random variation in the rate at which infection progresses. One hundred replicate simulations were run for each of the 42 plausible parameter sets (see Methods for further details)

The most plausible models suggest that there is little effect of variation in dispersal on disease outcomes. Neither the median population size after 10 years nor the median proportion of hibernacula with extant populations showed clear differences between the base model with philopatry of 0.96 and models with higher (0.92) and lower dispersal (1.0, i.e., perfect philopatry; Figure 5). Variation in hibernacula quality exhibited a different pattern, primarily as a broader prediction interval across simulations, with more instances of persistent populations and reduced extinction risk when the rate of progression of the disease (*γ*) varied between hibernacula (Figure 5).

## 4 DISCUSSION

The spread of white‐nose syndrome is an inherently multiscale process, but has been primarily studied at either the scale of an individual hibernaculum or the entire landscape. Our model explicitly linked the disease dynamics of individual hibernacula with the spread of WNS among little brown bat hibernacula. This approach provided better understanding of how aspects of disease dynamics affect regional population decline and persistence of little brown bats while assessing the uncertainty of predictions given current information. We explored a range of parameter values centered on empirically measured values for transmission, mortality, and other processes and found multiple plausible parameter sets (Drake et al., 2015). We found that multiple combinations of infection and dispersal processes can produce outcomes consistent with multiple observations of the epidemic. These plausible parameter combinations reproduce observed patterns in prevalence and the rate of cave infections, and simulations using these models predict a broad range of possible outcomes. These possible outcomes encompass both the dire predictions of metapopulation extirpation and possible species extinction (Frick et al., 2010) and more recent observations of colonies persisting at very low density (Frick et al., 2017; Langwig et al., 2017). The high variability in outcomes for individual parameter sets shows the important role of stochastic infection dynamics in determining the severity and rate of spread of the epidemic.

Our within‐hibernaculum model combines important aspects of WNS dynamics that have been explored individually in previous models, including *Pd* growth in the environment (Meyer et al., 2016; Verant et al., 2018) and multiple classes of infected individuals (Langwig et al., 2017), and adds to these models by incorporating differential shedding and infectivity between classes of infected bats. Importantly, we also consider how density‐dependent transmission and frequency‐dependent transmission between bats alter infection dynamics. Early results suggested that WNS‐driven declines depended on bat colony density for tricolored and northern long‐eared bats (Langwig et al., 2012). In contrast, the clumping behavior of hibernating little brown bats and lack of evidence for density dependence of disease dynamics in this species (Langwig et al., 2012) has led recent models to assume that disease transmission in little brown bats is independent of density (Langwig et al., 2017; Meyer et al., 2016). Our results suggest that frequency‐dependent transmission is consistent with the epidemic patterns for a much broader range of transmission parameters, including recent empirical estimates (Langwig et al., 2017). Density‐dependent transmission at medium to high values almost universally resulted in 100% prevalence in the first year of infection, diverging from observations (Frick et al., 2017). It also resulted in cases of epidemic fade‐out that are not currently matched by observation. Therefore, density‐dependent formulation of transmission leads to more extreme outcomes due to potential for population persistence following infection fade‐out or burnout, but also the possibility of more rapid initial declines (Lloyd‐Smith et al., 2005). Based on these divergent outcomes, we advocate that future studies aim to further investigate differences in mode of transmission between species, as well as how transmission modes could drive differences in extinction risk (Ryder, Miller, White, Knell, & Boots, 2007). Multiple *Pd*‐susceptible species often cohibernate, but our model does not consider these interactions because of the limited knowledge of *Pd* infection dynamics and bat population dynamics for species other than the little brown bat. More empirical data on other species could elucidate the potential interspecific differences in transmission pattern, which could drive transmission differences based on the presence of multiple species in hibernaculum (Hoyt et al., 2018). These possible differences have also likely increased the noise in the empirical observations to which we have compared these model results for the little brown bat.

At the among‐hibernacula scale, we model the spread of infection mechanistically, via a process where infection occurs as bats enter and leave hibernacula during fall swarming. This is the first comparison of this hypothesis to data, and we find this mechanism can be consistent with the spread of WNS in New York. Additionally, our model is novel in including stochastic dynamics and considering the impact of bat population size and site fidelity on disease and population dynamics. Our results provide support for fall swarming as a plausible mechanism of among‐hibernacula spread; cave density (a proxy for population size) has been shown to matter in disease transmission (Maher et al., 2012), which is consistent with the positive relationship between among‐hibernacula spread and bat population size following the process we model here. However, the actual process of hibernaculum‐to‐hibernaculum transmission by bats is still under study and additional field measurements are necessary to further determine whether multiple mechanisms contribute to among‐hibernacula spread of *Pd*. One possible explanation is the one we model here, where caves are infected by bats that acquire *Pd* from a previously infected hibernaculum during fall swarming (or summer roosting (Ballmann et al., 2017)), then develop infection in their previously uninfected hibernaculum. Alternatively, bat population density may be unrelated to among‐hibernacula infection, and spread may depend only on the density of infected hibernacula (O'Regan et al., 2015). These possibilities may be able to be distinguished through additional direct field measurements and validation of this model against a more precise metric of hibernacula infection rate.

Another key aspect of bat biology that we consider in the multiscale model is the tendency of bats to return to the same hibernaculum across years. High site fidelity has been observed in little brown bats (Kunz, 1982; Norquay et al., 2013), but the amount of variance and its source, for example, due to differences between species or environments, is not well understood. While it has been suggested that fidelity varies geographically, this and any other model will be limited by the paucity of published information on philopatry. The degree of fidelity is important to the realized rate of decline, since infection of the hibernacula with largest colonies will lead to rapid loss of bats, but we found that increasing or decreasing rates of hibernaculum fidelity did little to change hibernacula infection rates or metapopulation decline under the transmission mechanism we model here.

The model analyzed here differs from previous models in the inclusion of stochasticity at both the within‐hibernaculum and among‐hibernacula scales. Our results confirm that stochasticity in infection dynamics within hibernacula can lead to substantial departures from the deterministic case by slowing the initial rate of infection. Previous models have been partially or fully deterministic. Meyer et al. (2016) used a deterministic model of infection and population dynamics in a single hibernaculum to understand the importance of environmental persistence of *Pd*; an earlier model connecting among‐hibernacula dynamics to macroscale spread (O'Regan et al., 2015) considered stochasticity at the continental scale, but treated local hibernaculum‐to‐hibernaculum dynamics as a deterministic process. The results here demonstrate that stochasticity in bat infection has the potential to accelerate initial infection in a hibernaculum and that these within‐hibernaculum dynamics tend to alter rates of spread among hibernacula. The effect on survival is dependent on the model parameters but can also deviate substantially from the deterministic assumption.

By linking infection dynamics within multiple hibernacula, we were able to consider the effect of among‐hibernacula heterogeneities on disease transmission and population persistence. We found that including variation in hibernaculum quality had small but positive effects on bat populations, where average final population sizes were higher and the proportion of hibernacula populations extinct was lower when hibernacula were assumed to vary in their quality. Multiple empirical studies indicate that microclimate strongly influences *Pd* growth, with warmer and more humid sites increasing fungal load and mortality (Langwig et al., 2012; Wilder, Frick, Langwig, & Kunz, 2011). While some of these differences may be confounded by species differences (Moore et al., 2018; Wilder et al., 2011; Willis, Menzies, Boyles, & Wojciechowski, 2011), models and laboratory studies suggest that effects of temperature on *Pd* and bat energetics could strongly influence mortality (Boyles & Willis, 2010; Forsythe et al., 2018; Hallam & Federico, 2012; Marroquin, Lavine, & Windstam, 2017). Therefore, natural differences among hibernacula, coupled with metapopulation dynamics, could substantially slow spread and/or population decline. Our model suggests that rescue effects or refuges from WNS could promote bat survival. However, more work is needed to quantify the actual variation in hibernaculum quality. Additionally, there may be interactions (positive or negative) between the predisease number of bats in a cave and microclimate conditions affecting fungal growth and transmission.

Data on many aspects of WNS transmission are sparse, making parameter estimation for complex models difficult. Previous models of WNS have used various methods, including maximum likelihood (Langwig et al., 2017) and literature values (Meyer et al., 2016) for parameterization, but all approaches have been limited by data availability. The problem of fitting is intensified when considering multiple scales that have not been previously integrated. This is why we used the method of plausible parameter sets (Drake et al., 2015). Using this method, we found that no model outputs matched all the observed metrics, while the matching parameters also had flat distributions within the ranges tested. The latter suggests the reported values for these parameters fall in the center of a plausible range, and our difficulty in matching the empirical data has several likely explanations. First, the model may miss certain aspects of disease transmission, particularly in the poorly understood process of among‐hibernacula spread. This challenge is highlighted by the small number of single parameter sets that matched the observed number of newly infected caves in both years 2 and 3. However, this problem could be equally due to issues of detectability because *Pd* presence is often not visually detectable, particularly recently after a hibernaculum becomes infected (Janicki et al., 2015). This imperfect detection likely results in some inaccuracy in the empirical data from New York. For instance, a one‐year delay in detection would produce a slower apparent spread of the disease, and could appear as an acceleration in infection if the rate of imperfect detection declined over time. However, given the stage of the epidemic, consistent detection methods, spatial scale, and relevant estimate of little brown bat metapopulation size, these New York data represent the best existing proxy for a time series of hibernaculum‐level *Pd* spread. The accuracy of these data improves over time due to advances in survey methods and detection techniques, which will result in more precise estimates of *Pd* arrival in hibernacula. It is also the case that estimates for some parameters are limited and that their uncertainty is ill‐defined. One limitation was data on within‐hibernaculum fungal growth; to our knowledge, there are no existing datasets that include combined measurements of *Pd* dynamics in the environment and multistage infection dynamics (Reynolds et al., 2015). Furthermore, fungal measurements in the environment often cannot easily be converted across scales, as *Pd* from the environment has been measured as presence/absence (Langwig, Hoyt, et al., 2015), grams (Frick et al., 2017), or DNA abundance (Verant et al., 2018). Given limited data, cross‐scale feedbacks, and the stochasticity in the model, plausible parameter sets provided an appropriate way for us to understand the range of possible dynamics and the sensitivity of the model to specific parameters (Drake et al., 2015).

The outcomes of this model generally match the bleak outlook for little brown bat populations from previous studies (Frick et al., 2010; Maher et al., 2012; O'Regan et al., 2015). The metapopulation size is drastically reduced by year 10 in nearly all of the 42 most plausible scenarios, with an median decline of 98.6% and total extinction in 67% of cases (extinction is a decline below 0.01% of the initial population; (Frick et al., 2010)). However, some plausible parameter sets do allow for persistence of bats over this 10‐year period, particularly in the presence of potential rescue effects from variation in hibernacula climate across the landscape or stochastic fade‐out. The wide range of potential predictions, particularly in hibernaculum occupancy, supports the need for models like this one that consider the metapopulation dynamics of bat population declines due to WNS.

Alternatively to the hibernaculum climate hypothesis, little brown bats may be able to persist in infected hibernacula through the evolution of resistance to the fungus ( Langwig et al., 2017). Evidence shows that surviving bats in persistent hibernacula are infected with *Pd*, but maintain relatively low loads, and as a result can survive the winter (Frick et al., 2017; Langwig et al., 2017). This is consistent with independent work finding increased survival over time in little brown bats (Maslo et al., 2015). Our model does not include the possibility of resistance. We chose not to include resistance because our primary goal was to understand whether density dependence and metapopulation dynamics could result in a similar pattern of persistence. However, it is possible that the combination of the factors we explored, alongside evolution of resistance (Maslo & Fefferman, 2015), could further promote survival and persistence of bats in WNS‐infected caves. Future studies of WNS that consider these processes in combination will be important to determine the relative importance of immunity and metapopulation dynamics in allowing persistence of bats into the future, particularly as time series on bat population sizes grow.

By linking the within‐hibernaculum and between‐hibernacula scales, we have been able to gain insight into the spread of WNS and the role of local‐scale and metapopulation dynamics in determining the spread of the disease and the decline of North American bat species. Previous models have not connected these two scales or considered the stochastic dynamics likely to be important in these processes. Thus, our model provides a way to evaluate scenarios of future bat declines, including time to extinction. Furthermore, it allows for comparison of competing explanations for the persistence of bat colonies in the wake of WNS‐induced declines and a flexible structure for incorporating new evidence for the mechanisms of WNS transmission. Bat species differ in sociality, preferred hibernation habitat, and impacts of WNS (Langwig et al., 2012), but this model is generalizable to other bat species, since similar mechanisms of transmission and spread apply across species. Considering of these alternative possibilities will inform forecasts of population declines and priorities for future field and laboratory studies of bat behavior, disease progression, and fungal biology.

## CONFLICT OF INTEREST

None declared.

## AUTHOR CONTRIBUTIONS

AK, CT, and AG conceived the ideas; AK, CT, AG, and JD designed methodology; AK, CT, and AG wrote simulation code and analyzed data; AK and CT led the writing of the manuscript. All authors contributed critically to the drafts and gave final approval for publication.

## Data Availability

Code is archived at Dryad along with summarized simulation output data, https://doi.org/10.5061/dryad.nr75483 (Kramer, Teitelbaum, Griffin, & Drake, 2019) Full simulation output data are too large to archive, but can be recreated using the simulation code.
