# Supplementary material for: Multiscale model of regional population decline in little brown bats due to white‐nose syndrome
Source: Ecol Evol. 2019 Jul 4;9(15):8639–51. doi: 10.1002/ece3.5405 (PMC6686297; doi:10.1002/ece3.5405)

Appendix S1

Multi-scale model of regional population decline in little brown bats due to white-nose syndrome

Andrew M. Kramer, Department of Integrative Biology, University of South Florida

Claire S. Teitelbaum, Odum School of Ecology, University of Georgia

Ashton Griffin, Odum School of Ecology, University of Georgia

John Drake, Odum School of Ecology and Center for Ecology of Infectious Diseases, University of Georgia

Appendix S1 Table 1: Parameter values for the 42 best-fit parameter sets

ID	ϕ	ϵ	ψ	κ	$\beta1$	$\beta2$	$\gamma1$	$\gamma2$	μ	α	V	d
1	124.5	0.1172	0.1623	0.1	1.10E-13	2.78E-4	0.0122	0.0852	0.0408	0.99984	5.195	0
2	54.8	0.3804	0.2217	0.1	9.50E-12	1.20E-5	0.0155	0.0581	0.0200	0.99966	13.203	0
3	83.7	0.6167	0.0252	0.1	4.96E-12	3.47E-5	0.0182	0.0808	0.0426	0.99972	12.174	0
4	110.8	0.9604	0.0229	0.1	3.32E-12	7.69E-5	0.0134	0.0451	0.0311	0.99979	11.796	0
5	125.0	0.2767	0.1503	0.1	4.82E-13	1.64E-4	0.0186	0.0862	0.0275	0.99967	10.580	0
6	100.4	0.2340	0.0430	0.1	4.22E-12	6.44E-5	0.0164	0.0666	0.0913	0.99963	11.367	0
7	186.5	0.7619	0.0608	0.1	1.49E-13	3.72E-5	0.0175	0.0692	0.0807	0.99959	8.295	0
8	161.4	0.4565	0.1965	0.1	1.38E-13	3.14E-5	0.0136	0.0805	0.0666	0.99954	7.840	0
9	152.0	0.9107	0.1785	0.1	1.61E-13	2.47E-5	0.0160	0.0645	0.0310	0.99953	4.334	0
10	91.5	0.2994	0.1288	0.1	1.39E-12	2.64E-4	0.0200	0.0360	0.0397	0.99964	9.394	0
11	116.0	0.1208	0.1391	0.1	8.39E-12	2.38E-4	0.0153	0.0332	0.0998	0.99995	9.535	0
12	26.4	0.3680	0.1655	0.1	1.68E-13	1.14E-4	0.0160	0.0197	0.0464	0.99980	10.525	0
13	78.2	0.4944	0.1273	0.1	2.66E-12	1.58E-4	0.0168	0.0937	0.0928	0.99967	7.068	0
14	121.9	0.1402	0.2012	0.1	6.36E-13	3.27E-5	0.0137	0.0316	0.0403	0.99965	12.814	0
15	184.5	0.1021	0.1808	0.1	3.83E-13	7.48E-5	0.0131	0.0727	0.0954	0.99957	9.722	0
16	163.3	0.9314	0.3553	0.1	2.38E-13	2.47E-2	0.0103	0.0983	0.0630	0.99968	8.478	0
17	19.6	0.0887	0.2705	0.1	6.41E-12	1.39E-5	0.0130	0.0454	0.0477	0.99974	8.744	0
18	54.7	0.6114	0.1631	0.1	1.20E-12	3.07E-5	0.0192	0.0569	0.0135	0.99980	11.478	0
19	31.7	0.1916	0.1590	0.1	8.45E-13	5.44E-5	0.0142	0.0644	0.0934	0.99968	6.977	0
20	145.5	0.8535	0.0460	0.1	2.82E-12	1.60E-5	0.0172	0.0603	0.0364	0.99956	9.800	0
21	16.8	0.1414	0.3070	0.1	2.15E-12	4.01E-5	0.0131	0.0469	0.0430	0.99966	7.379	0
22	1007	0.7879	0.2842	0.1	6.85E-12	1.52E-3	0.0177	0.0519	0.0907	0.99978	11.088	1
23	185.7	0.0763	0.2117	0.1	2.23E-13	3.30E-5	0.0104	0.0800	0.0723	0.99996	12.969	1
24	151.4	0.1164	0.2438	0.1	3.96E-13	7.68E-2	0.0189	0.0397	0.0210	0.99987	12.416	1
25	81.0	0.7864	0.2386	0.1	2.80E-12	4.17E-2	0.0142	0.0150	0.0308	0.99988	6.034	1
26	54.8	0.3804	0.2217	0.1	9.50E-12	1.20E-5	0.0155	0.0581	0.0200	0.99966	13.203	1
27	60.0	0.4575	0.1119	0.1	3.43E-13	9.37E-2	0.0192	0.0794	0.0509	0.99973	7.482	1
28	108.8	0.1674	0.3454	0.1	7.05E-13	6.57E-2	0.0158	0.0950	0.0617	0.99985	13.105	1
29	177.1	0.1906	0.2261	0.1	3.07E-12	3.27E-2	0.0107	0.0130	0.0406	0.99985	12.317	1
30	170.7	0.0622	0.2500	0.1	3.94E-12	1.02E-1	0.0178	0.0549	0.0419	0.99982	10.969	1
31	49.5	0.1191	0.0820	0.1	1.34E-12	5.19E-2	0.0193	0.0474	0.0247	0.99951	4.554	1
32	37.1	0.0593	0.2232	0.1	1.17E-12	1.29E-1	0.0196	0.0598	0.0303	0.99959	4.796	1
33	156.6	0.8973	0.1900	0.1	1.21E-12	2.95E-4	0.0140	0.0310	0.0863	0.99971	1.869	1
34	179.5	0.1422	0.2077	0.1	4.81E-12	1.60E-1	0.0104	0.0767	0.0420	0.99976	6.475	1
35	87.8	0.9993	0.1600	0.1	8.22E-13	5.19E-2	0.0185	0.0609	0.0132	0.99969	6.511	1
36	50.8	0.5340	0.1779	0.1	2.89E-12	9.56E-2	0.0198	0.0943	0.0818	0.99966	1.697	1
37	89.8	0.8883	0.2096	0.1	9.71E-12	5.23E-3	0.0162	0.0307	0.0257	0.99976	7.434	1
38	145.3	0.5190	0.3408	0.1	1.25E-12	4.86E-2	0.0188	0.0435	0.0942	0.99951	4.608	1
39	32.6	0.1856	0.2038	0.1	1.03E-13	1.13E-1	0.0172	0.0607	0.0594	0.99995	13.059	1
40	165.2	0.8111	0.3257	0.1	2.94E-12	1.98E-3	0.0122	0.0840	0.0707	0.99960	5.573	1
41	160.7	0.3236	0.1855	0.1	1.05E-13	7.39E-2	0.0121	0.0321	0.0273	0.99977	8.076	1
42	139.1	0.7289	0.1360	0.1	1.18E-12	5.78E-2	0.0156	0.0753	0.0219	0.99958	4.411	1

Appendix S1 Figure 1

Match of model output with 8 goodness of fit measures plotted against individual parameter values. Each point is a parameter combination with the line representing the 95% prediction interval from the set of 100 simulations of each parameter combination. Goodness of fit measures are represented by red lines or regions. The upper bound on environmental *Pd* is truncated to improve readability and focus on the relevant range of possibilities. Both frequency dependent and density dependent outcomes are shown, resulting in overlapping lines at each parameter value.

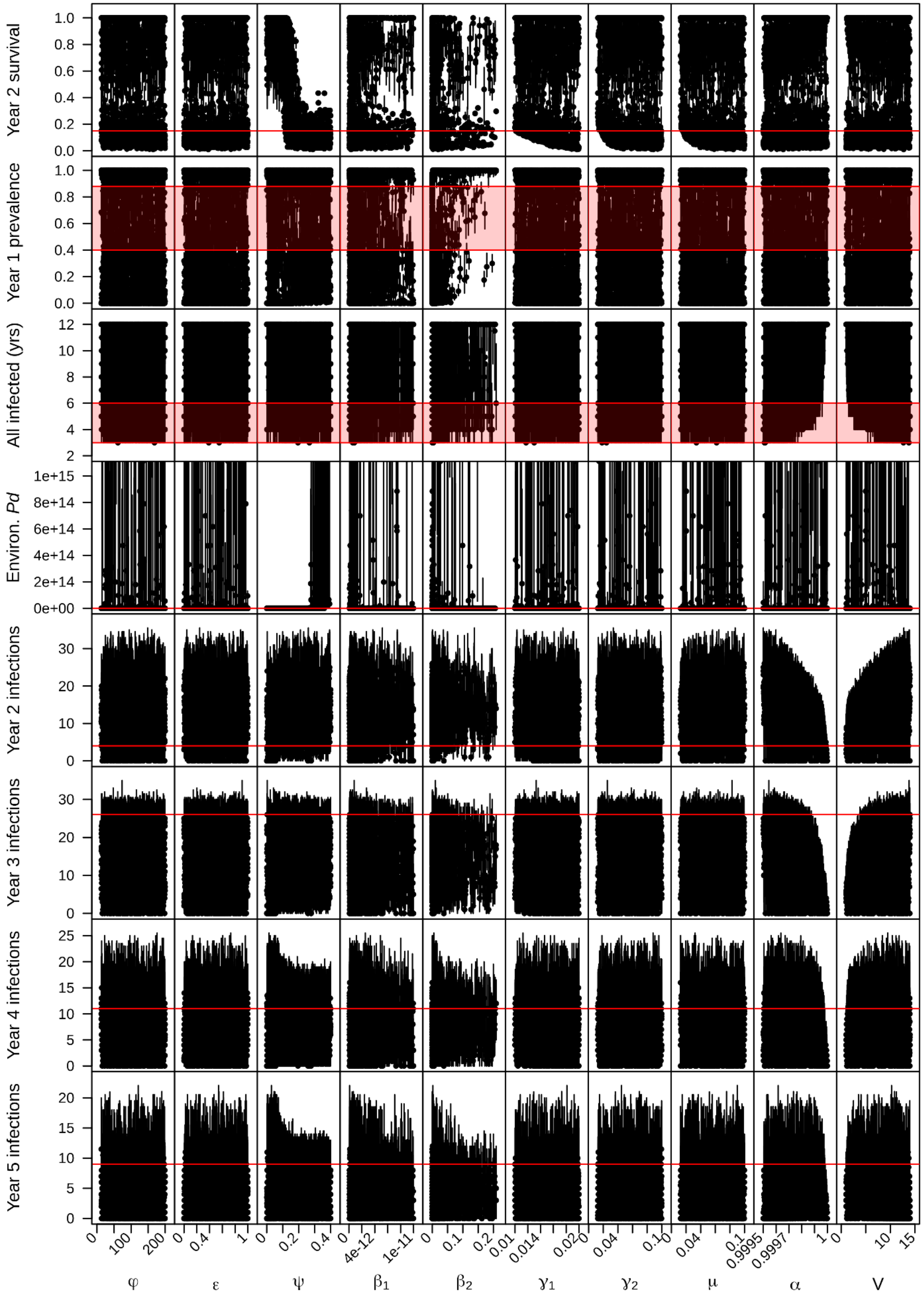

Year 2 survival
Year 1 prevalence
All infected (yrs)
Environ. Pd
Year 2 infections
Year 3 infections
Year 4 infections
Year 5 infections
φ
ε
ψ
β1
β2
γ1
γ2
μ
α
V

Appendix S1 Figure 2

Match of model output with 8 goodness of fit measures plotted against individual parameter values for those parameter combinations including frequency dependent transmission. Each point is a parameter combination with the line representing the 95% prediction interval from the set of 100 simulations of each parameter combination. Goodness of fit measures are represented by red lines or regions. The upper bound on environmental *Pd* is truncated to improve readability and focus on the relevant range of possibilities.

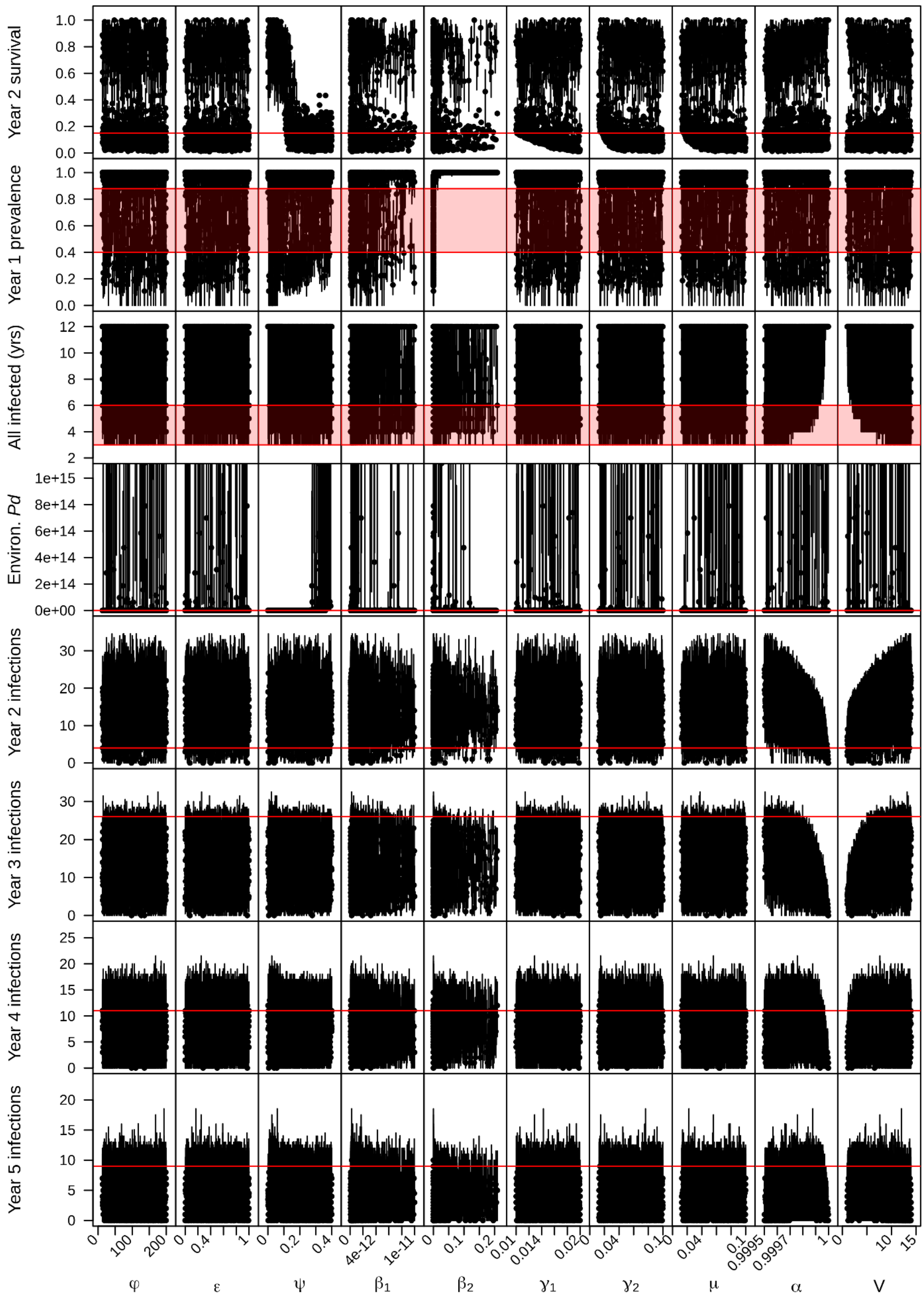
Year 2 survival
Year 1 prevalence
All infected (yrs)
Environ. Pd
Year 2 infections
Year 3 infections
Year 4 infections
Year 5 infections
φ
ε
ψ
β1
β2
γ1
γ2
μ
α
V

Appendix S1 Figure 3

Match of model output with 8 goodness of fit measures plotted against individual parameter values. Each point is a parameter combination with the line representing the 95% prediction interval from the set of 100 simulations of each parameter combination. 42 combinations match 6 of 8 and an additional 330 include 5 of 8 (372 total combinations shown). Goodness of fit measures are represented by green lines or regions. The points in orange are combinations with frequency dependent transmission, those in blue had density dependent transmission.

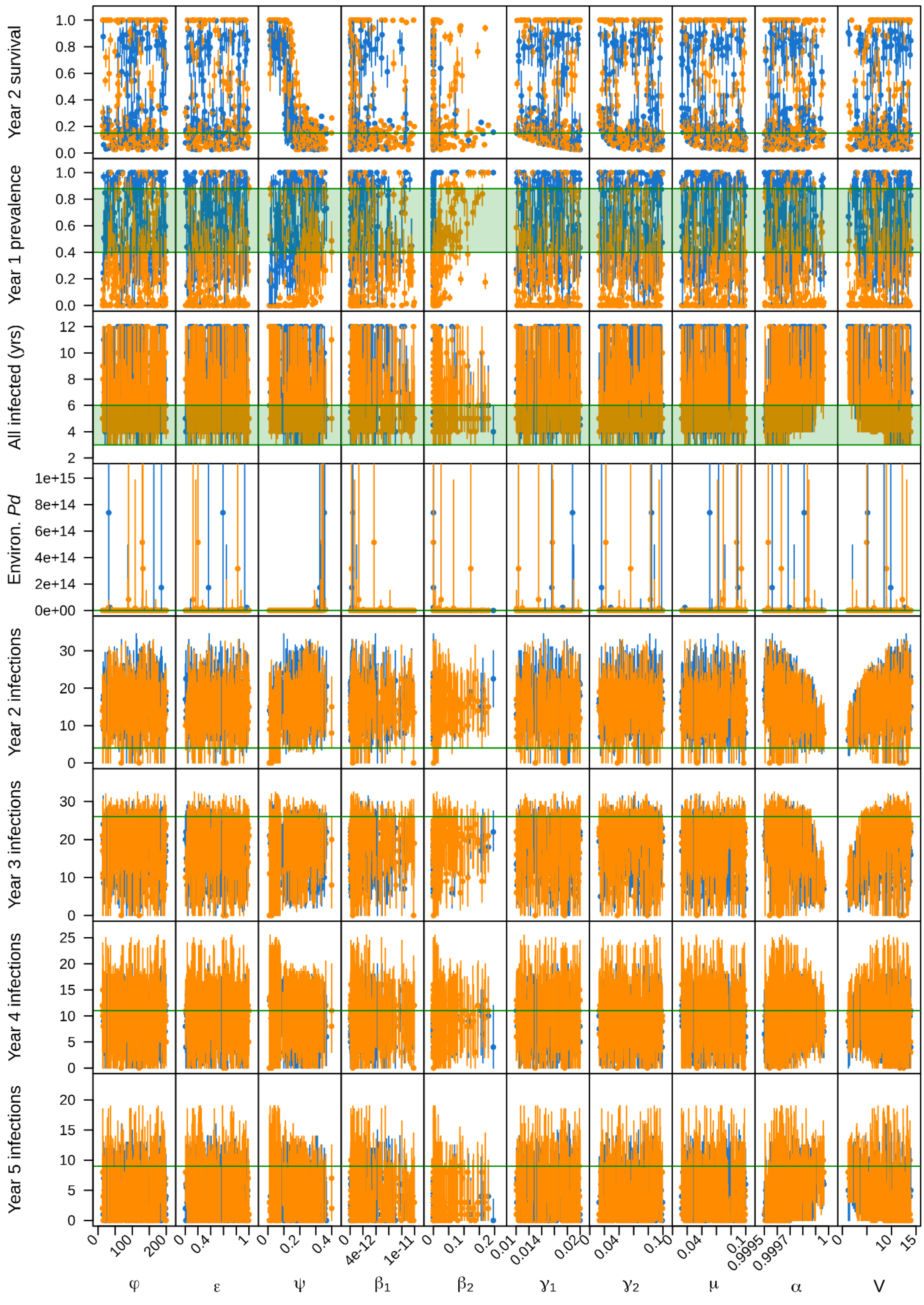
Year 2 survival
Year 1 prevalence
All infected (yrs)
Environ. Pd
Year 2 infections
Year 3 infections
Year 4 infections
Year 5 infections
φ
ε
ψ
β1
β2
γ1
γ2
μ
α
V

Appendix S1 Figure 4

Match of model output with 8 goodness of fit measures plotted against individual parameter values for those parameter combinations including density dependent transmission. Each point is a parameter combination with the line representing the 95% prediction interval from the set of 100 simulations of each parameter combination. Goodness of fit measures are represented by red lines or regions. The upper bound on environmental *Pd* is truncated to improve readability and focus on the relevant range of possibilities.

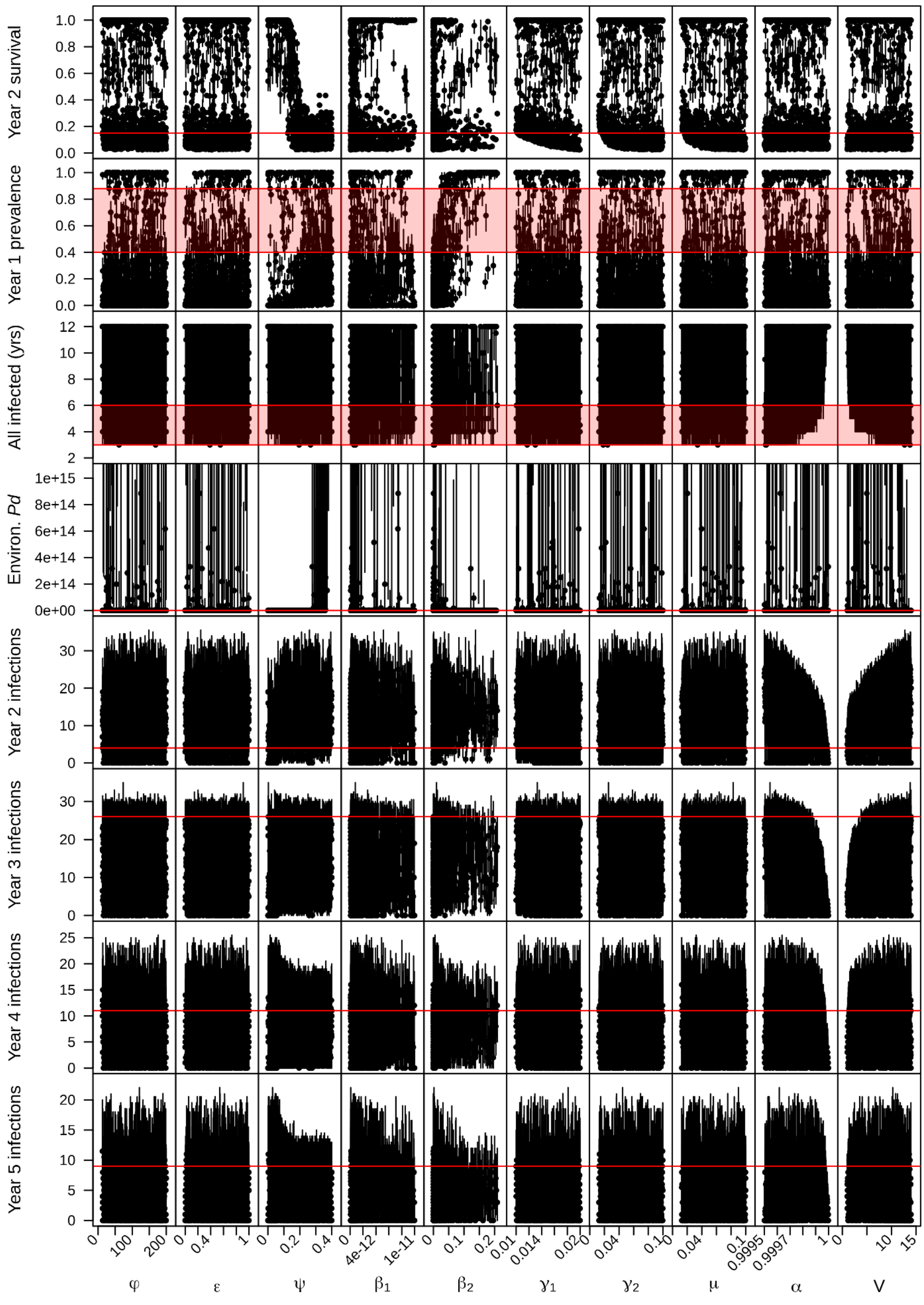
Year 2 survival
Year 1 prevalence
All infected (yrs)
Environ. Pd
Year 2 infections
Year 3 infections
Year 4 infections
Year 5 infections
φ
ε
ψ
β1
β2
γ1
γ2
μ
α
V

Appendix S1 Figure 5

Histogram of the parameter values from the extended plausible parameter sets. Included are parameter values from all combinations that matched 5 or more of the 8 goodness of fit measures. Red lines indicate the median value.

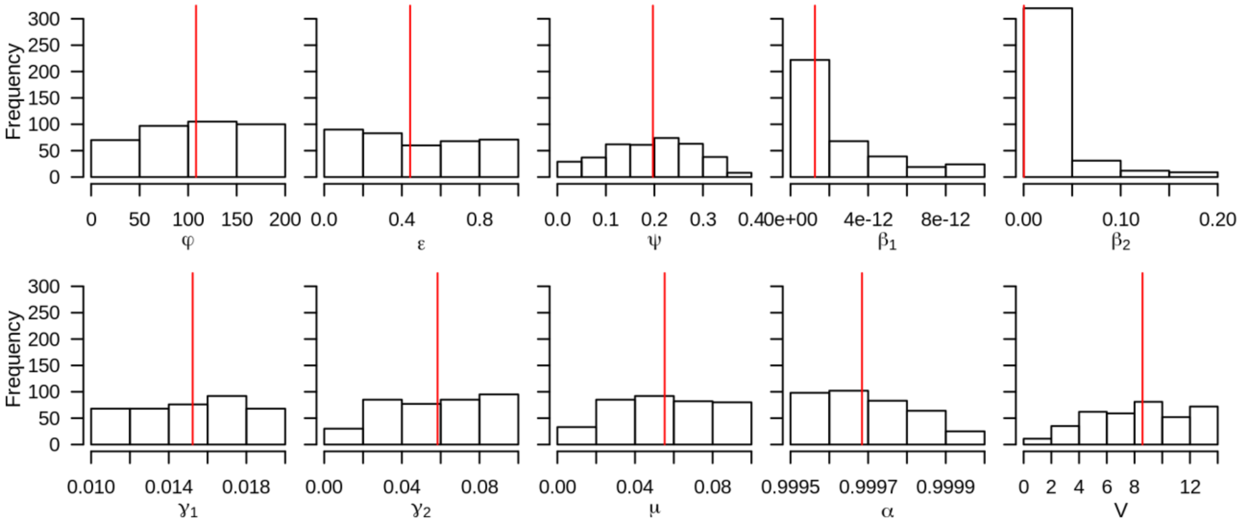

Appendix S1 Figure 6

Correlations between pairs of parameters for the plausible parameter sets. The value in bold represents significant correlation after correcting for multiple tests.

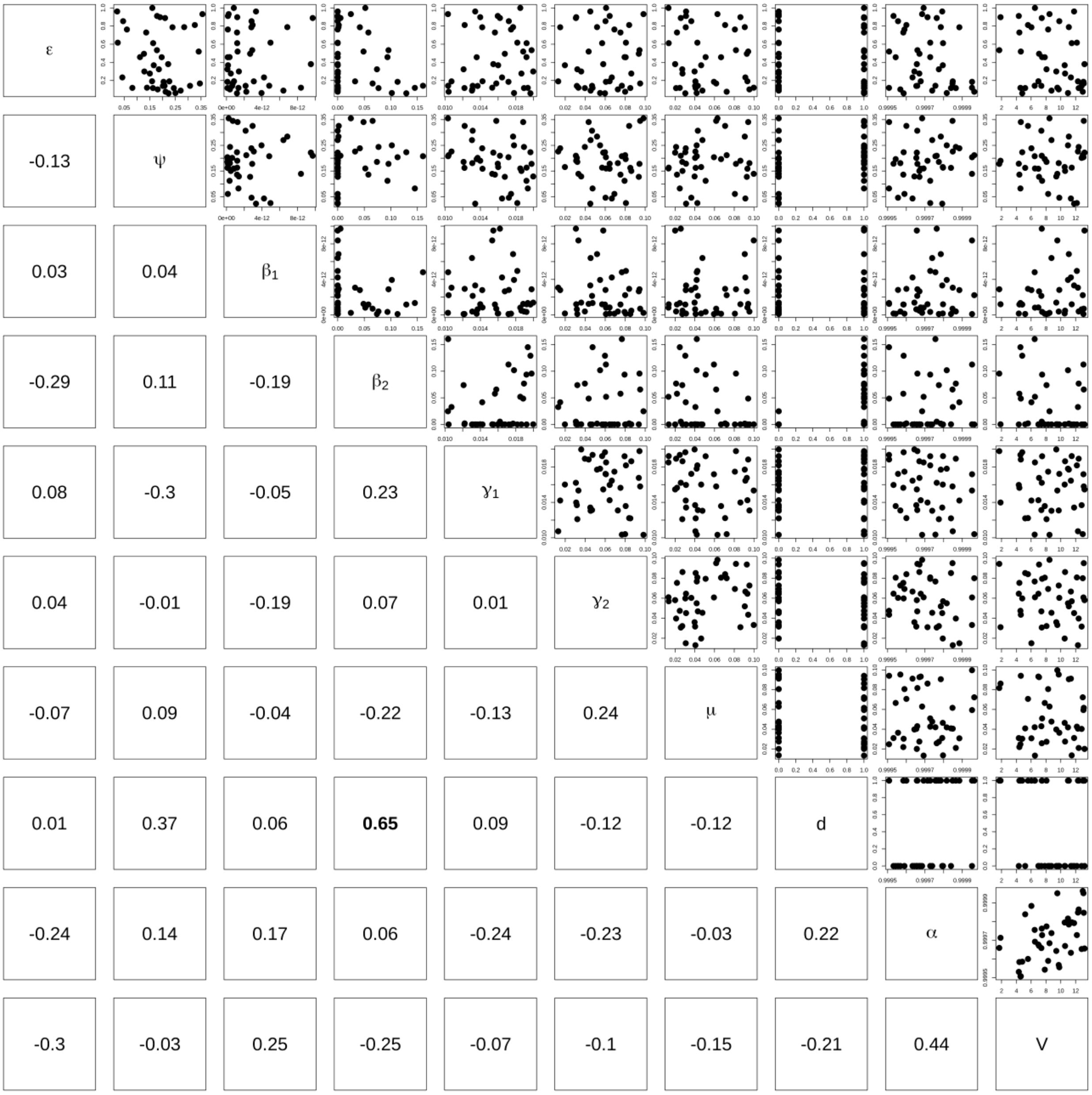

Appendix S1 Figure 7

Trajectories of the population of each cave for replicate simulations of the plausible parameter sets. Lines from each simulation of each parameter set are overlain. The density-dependent parameter sets are blue and frequency dependent transmission is in orange. The fixed points at time zero represent the shared initial population sizes of each cave.

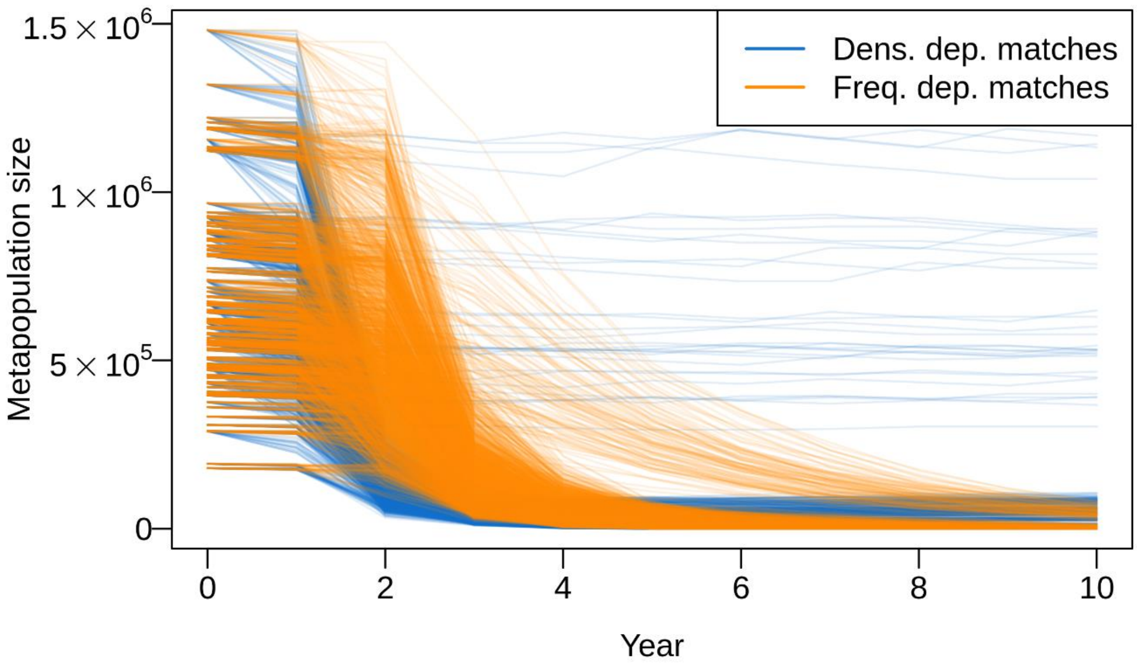

Appendix S1 Figure 8

Trajectories of the number of caves infected each year for replicate simulations of the plausible parameter sets. Lines from each simulation of each parameter set are overlain. The density-dependent parameter sets are blue and frequency dependent transmission is in orange.

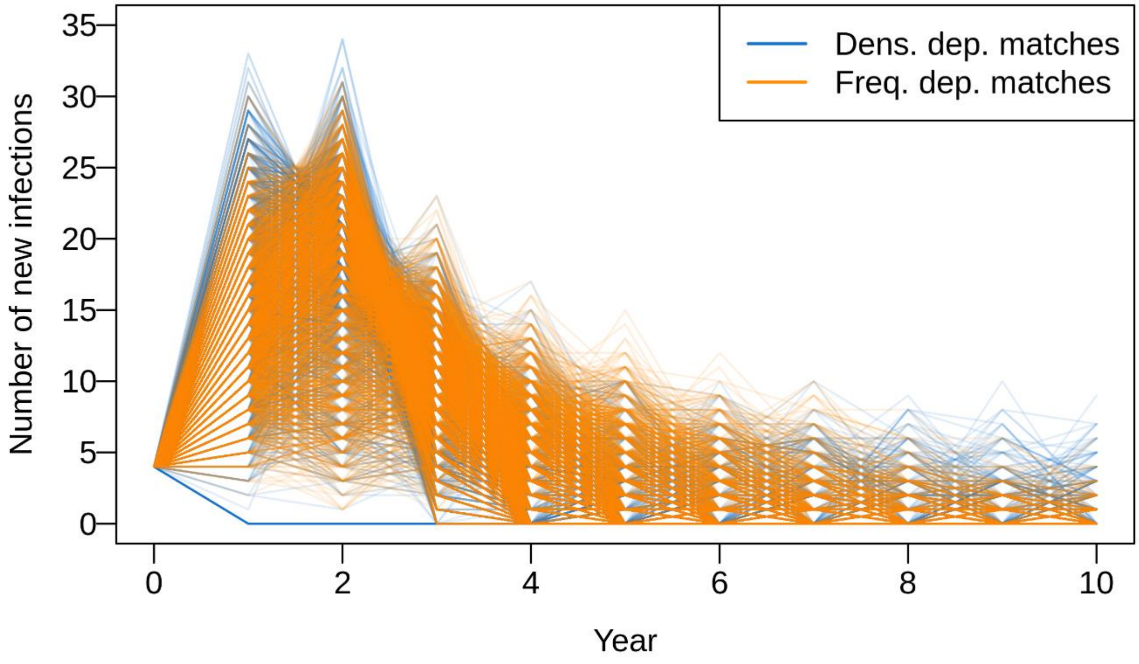

Appendix S1 Figure 9

Correspondence in cave-level survival in year 2 between stochastic and deterministic simulations for each parameter combination. Dots are medians and lines are the 95% prediction intervals. Variation in deterministic simulations comes from differences in timing and order of cave infection.

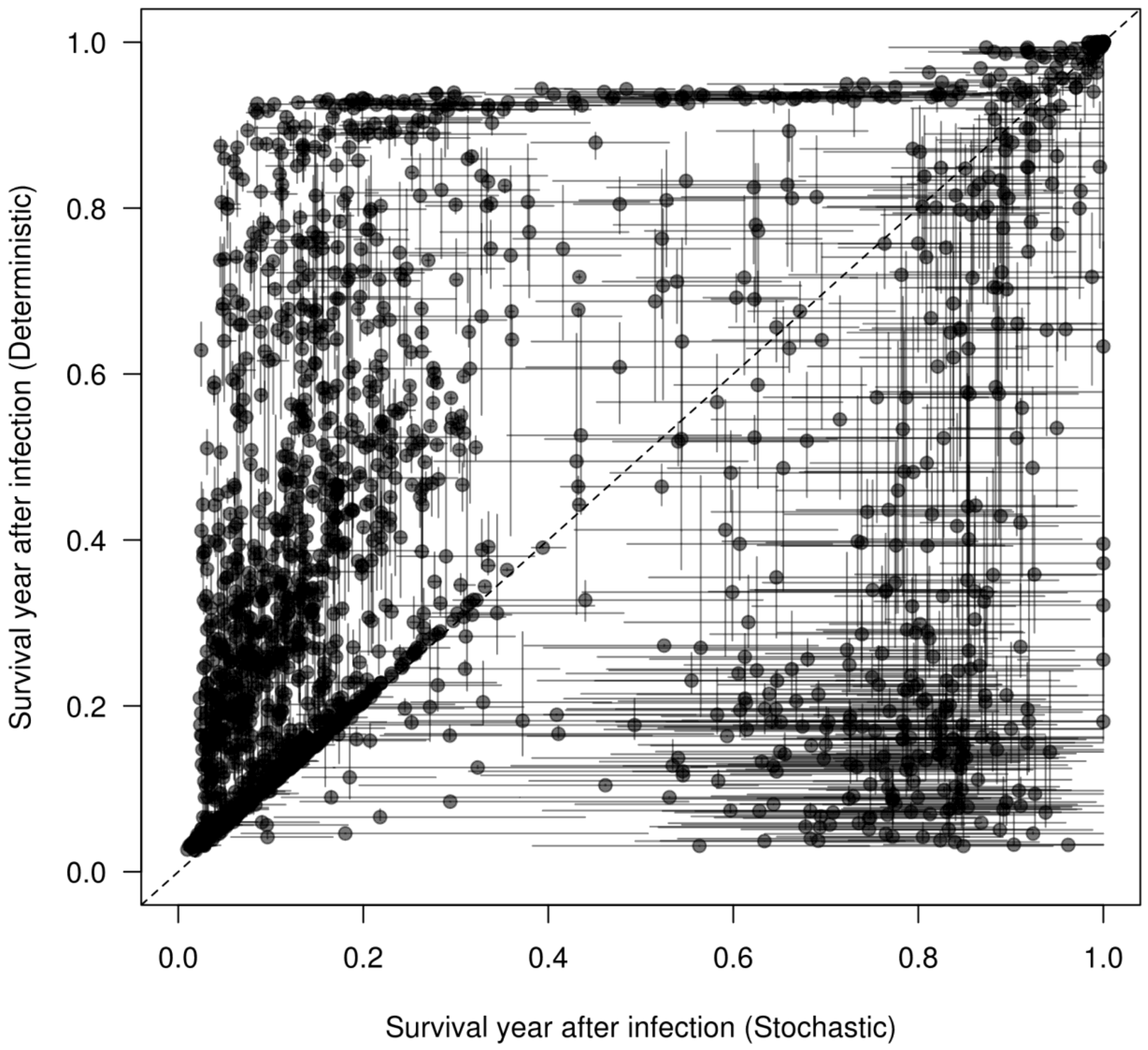

Appendix S1 Figure 10

Correspondence in number of caves infected in the second year between stochastic and deterministic simulations for each parameter combination. Dots are medians and lines are the 95% prediction intervals. Variation in deterministic simulations comes from differences in timing and order of cave infection.

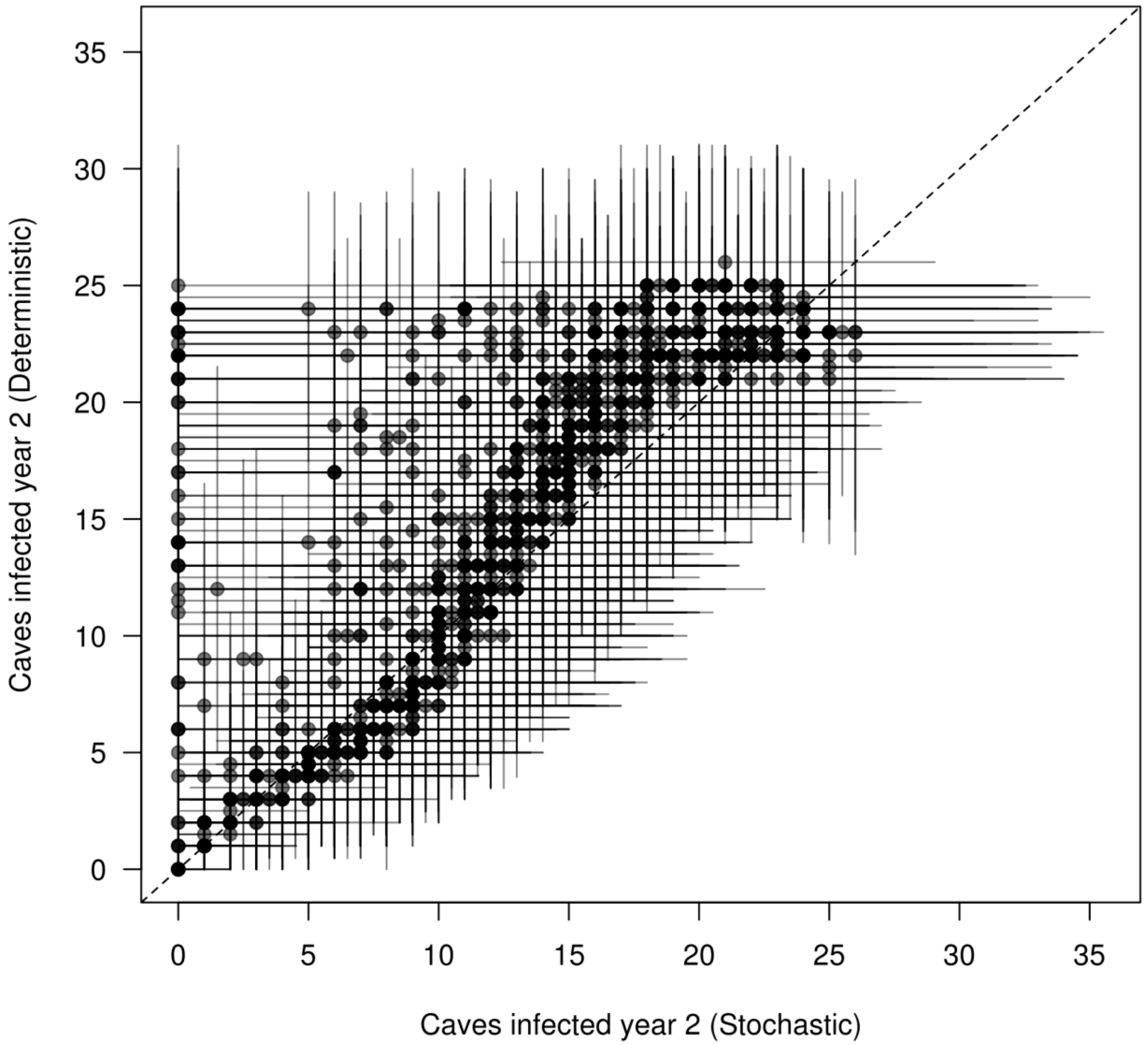

Supplement: Supplementary file 1 [file ECE3-9-8639-s001.pdf]
